# Effects of Atomic Ratio on the Mechanical Properties of Amorphous Silicon Carbon Nitride

**DOI:** 10.3390/ma15196865

**Published:** 2022-10-02

**Authors:** Chaoyue Ji, Xintian Cai, Zhen Zhou, Bing Gao, Sheng Liu

**Affiliations:** 1 The Institute of Technological Sciences, Wuhan University, Wuhan 430072, China; 2 School of Power and Mechanical Engineering, Wuhan University, Wuhan 430072, China; 3 School of Mechanical Science & Engineering, Huazhong University of Science & Technology, Wuhan 430074, China

**Keywords:** mechanical properties, molecular dynamics, silicon carbon nitride, structural characterization

## Abstract

This paper evaluates the mechanical properties of amorphous silicon carbon nitride (a-SiC${}_{x}$N${}_{y}$) films with different atomic ratios via molecular dynamics simulation. The Si-C-N ternary amorphous model is constructed using ReaxFF potential and melt-quenching method. The results demonstrate that the density range of constructed model spans a wide range of densities (2.247–2.831 g/cm^3^). The short- and medium-range order of the constructed a-SiC${}_{x}$N${}_{y}$ structures show a good correlation with the experimental observations. Based on the structural feasibility, the elastoplastic performance is analyzed. There is significant ductility during the uniaxial tension process of a-SiC${}_{x}$N${}_{y}$, except for Si(CN${}_{2}$)${}_{2}$. The calculated elastic modulus ranges from 206.80 GPa to 393.58 GPa, close to the experimental values of coating films. In addition, the elastic modulus of a-SiC${}_{x}$N${}_{y}$ does not change monotonically with the carbon or silicon content but is related to the atomic ratio. This article provides an understanding of the chemical composition dependence of the mechanical properties of amorphous compounds at the molecular level.

## 1. Introduction

Amorphous ternary silicon carbon nitride (a-SiC${}_{x}$N${}_{y}$) is widely used in optoelectronics, coatings, and semiconductor technologies due to its excellent properties, such as wide band gap, high-temperature stability, and outstanding mechanical properties [1,2,3]. The a-SiC${}_{x}$N${}_{y}$ films have received extensive attention as copper diffusion barriers and etching stoppers for multi-level interconnects in ultra large-scale integration (ULSI). The a-SiC${}_{x}$N${}_{y}$ films need to be as thin as possible to meet the demands of low R-C time delays and higher density devices, which pose challenges for mechanical reliability. Therefore, understanding the composition–structure–property relationship of a-SiC${}_{x}$N${}_{y}$ is a crucial issue.

The nanocrystalline SiCN film fabricated by reactive radio-frequency (RF) magnetron sputtering exhibited dielectric stability and temperature-invariant dielectric tunability at an elevated temperature of 673 K [4]. Through X-ray diffraction spectroscopy, Fourier transform infrared spectroscopy and photoluminescence, different nitrogen flow rates of plasma-enhanced chemical vapor deposition (PECVD) influenced the structural properties of SiCN films [5]. The mechanical properties were reported as follows. The effective modulus of hydrogen-free and hydrogen-containing a-SiC${}_{x}$N${}_{y}$ was 211–258 GPa and 115–144 GPa, respectively [6]. The flexural strength of the sintered a-SiC${}_{x}$N${}_{y}$ nanocomposite increased with the decrease in porosity [7]. The nanostructure of hydrogenated carbon nitride (CN${}_{x}$:H) thin films deposited by RF PECVD was sensitive to the N${}_{2}$:(N${}_{2}$ + CH${}_{4}$) flow rate ratio [8]. The mechanical properties depend on the element content and atomic arrangement, which strongly correlates with the material growth process parameters. It is difficult to systematically analyze the chemical composition dependence of the structural and mechanical properties of a-SiC${}_{x}$N${}_{y}$ using experimental methods. Molecular dynamics (MD) have been extensively used to calculate compound properties. MD was employed to analyze the effects of doping concentration and atomic number on the structural characteristics, phase transition, and crystallization of Fe${}_{1-x-y}$Ni${}_{x}$Co${}_{y}$ alloys [9]. The local atomic arrangement of ternary Al-Ni-Ti metallic glass was simulated based on TB-SMA potential [10]. Moreover, the MD results also facilitate macroscopic calculations based on finite element analysis (FEA) and develop experimental parameters as needed. FEA was used to predict metal-on-metal bearing contact pressure for three different metallic materials, namely cobalt chromium molybdenum (CoCrMo), stainless steel 316L (SS 316L), and titanium alloy (Ti6Al4V) [11]. There is a lack of theoretical research on the compound-structure-property relationship of a-SiC${}_{x}$N${}_{y}$, especially the microscopic mechanism of the effect of atomic ratio on mechanical properties.

In this paper, the structural properties and mechanical structural properties of a-SiC${}_{x}$N${}_{y}$ with different atomic ratios are estimated by the MD method. First, the structural configuration of a-SiC${}_{x}$N${}_{y}$ is generated using the ReaxFF potential and the melt-quenching method. Then, structural properties are obtained by radial distribution functions and structure factors, while mechanical properties are evaluated by Young’s modulus and Poisson’s ratio. The performance differences of a-SiC${}_{x}$N${}_{y}$ with different atomic ratios are discussed emphatically. Overall, this paper is devoted to quantitatively investigating the compositional dependence of the structure and properties of amorphous ternary silicon carbon nitride.

## 2. Simulation Methods

### 2.1. Preparation of the a-SiC${}_{x}$N${}_{y}$

**Figure 1:**
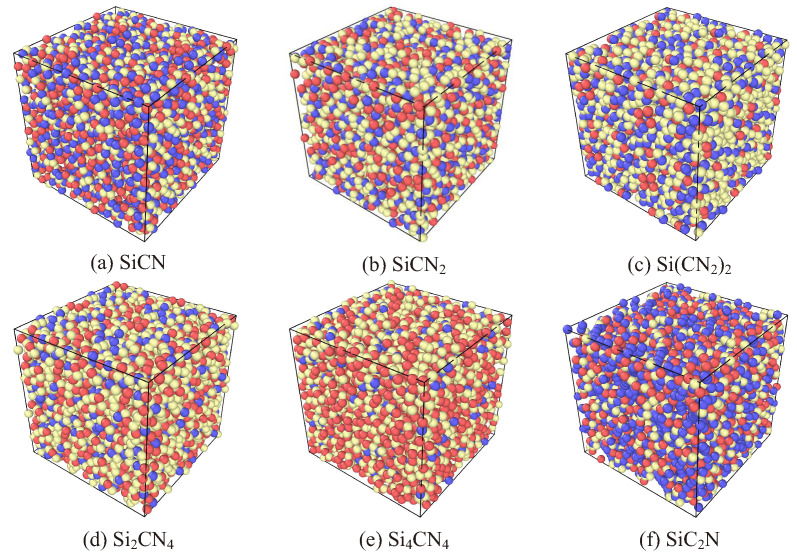
Schematic representation of a-SiC${}_{x}$N${}_{y}$ with different atomic ratios (Color scheme—Si: red; C: blue; and N: yellow).

The molecular structure of a-SiC${}_{x}$N${}_{y}$ is developed by following the general procedure of obtaining an amorphous structure from a random structure, called the melt-quench approach [12]. First, the initial structure of a-SiC${}_{x}$N${}_{y}$ is prepared by randomly placing the atoms (Si, C, and N) in a bounded box. The minimum distance between placed atoms is 2 Å. The structure is approximately 6100 atoms, and the ratio of Si, C and N atoms equal to 1:1:1, 1:1:2, 1:2:4, 2:1:4, 4:1:4, and 1:2:1, represents several silicon carbon nitrogen compounds as SiCN, SiCN${}_{2}$, Si(CN${}_{2}$)${}_{2}$, Si${}_{2}$CN${}_{4}$, Si${}_{4}$CN${}_{4}$, and SiC${}_{2}$N, as shown in Figure 1. In addition, models with randomly placed atoms of Si${}_{3}$N${}_{4}$ and SiC are generated for comparison.

**Figure 2:**
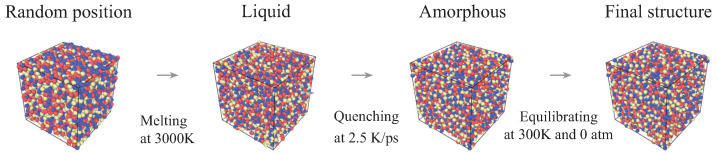
Schematic representation of model construction procedure. (Color scheme—Si: red; C: blue; and N: yellow).

The initial structures are melted at 3000 K to obtain a stable configuration, specifically, equilibrated for 50 ps in the canonical (NVT) ensemble and 150 ps in the isothermal-isobaric (NPT) ensemble. The structure decreases linearly from 3000 K to 300 K at a cooling rate of 2.5 K/ps. Such a cooling process is in an NPT ensemble, allowing the structure to shrink in response to the applied temperature. At a temperature of 300 K, the obtained structure is equilibrated for 40 ps in the NVT ensemble, followed by the NPT ensemble for another 40 ps before calculating properties. The constructed representative atomic structure of a-SiC${}_{x}$N${}_{y}$ with an atomic ratio of 1:1:1 is shown in Figure 2. The MD simulation is carried out with periodic boundary conditions (PBCs) in all three directions. All simulations are calculated by the open-source atomistic simulation code LAMMPS [13]. The interatomic interactions are modeled using a reactive force field (ReaxFF) potential [14] and the charge equilibration method [15]. A tolerance of $10^{-6}$ is adopted during charge equilibration. A time step equal to 0.5 fs is adopted. The thermodynamic properties are integrated using the Verlet method. Atomic configurations are visualized by OVITO [16].

### 2.2. Structural Characterization

Structural characterization is performed to evaluate the feasibility of the constructed amorphous SiC${}_{x}$N${}_{y}$ structures. The fundamental issue of analyzing the disordered phase is the specification of the network topology, which defines how atomic sites are connected. The a-SiC${}_{x}$N${}_{y}$ is a disordered system and its structure can be described by statistics, such as distribution functions. Thus, the Radial Distribution Functions (RDFs) give the density probability for an atom of the α species to have a neighbor of the β species at a given distance *r*, as shown in Equation (1) [17].

(1) $$g\left(r\right)=\frac{\sum_{\alpha ,\beta }c_{\alpha }b_{\alpha }c_{\beta }b_{\beta }g_{\alpha \beta }\left(r\right)}{\langle b^{2}\rangle }$$

where $c_{\alpha }$ is the concentration of atomic species α (α = Si, C, or N), g${}_{\alpha \beta }\left(r\right)$ are the partial RDFs, $b_{\alpha }$ is the neutron scattering length of the species, and $\langle b^{2}\rangle$ equal to ($\sum_{\alpha }^{}c_{\alpha }b_{\alpha }{)}^{2}$.

The total structure factor for the isotropic system is calculated based on the Fourier transform of the sum of partial radial distribution functions g${}_{\alpha \beta }\left(r\right)$. The structure factor is used to evaluate the medium-range characteristics of the atomic structure, which can be compared to experimental scattering data. The total scattering static structure factor is

(2) $$S\left(q\right)=1+4\pi \rho \int_{0}^{\infty }r^{2}\left[g(r)-1\right]\left(\frac{sin\left(qr\right)}{qr}\right)dr$$

where *q* represents scattering vector, $\rho_{0}$ is the average atom number density. The low-*q* limit of S(q) is defined by the box length L: $q_{min}=4\pi /L$.

### 2.3. Elastic Properties

The elastic properties of the constructed SiC${}_{x}$N${}_{y}$ structures are evaluated using the methodology described hereafter. Uniaxial tensile deformation is performed by progressive elongation to obtain the elastic properties of the a-SiC${}_{x}$N${}_{y}$. The tensile tests are simulated by applying uniaxial strain along the y-axis on the relaxed model (approximately 400,000 atoms) using a constant strain rate 0.001 ps${}^{-1}$. All simulations are carried out at 300 K and zero pressure using a Nose/Hoover barostat. The corresponding engineering strain is estimated by comparing the boundary distance of the tensile direction of the system. Specifically, the strain ε is calculated from the current length *L* and the original length $L_{0}$ of the bounded box: $\varepsilon =\left(L-L_{0}\right)/L_{0}$. The stress is computed using the Virial theorem. The Virial stress is represented by Equation (3) [18].

(3) $$\sigma_{ij}^{V}=-\frac{1}{V}\sum_{a=1}^{N}\left[m_{a}v_{i}^{\alpha }v_{j}^{\alpha }+\frac{1}{2}\sum_{\beta \neq \alpha }r_{ij}^{\alpha \beta }f_{ij}^{\alpha \beta }\right]$$

where the *V* represents the volume of the bounded box, *N* is the number of atoms, *m* is the atomic mass, *v* is the atomic velocity, *r* is the interatomic distance, and *f* is the interatomic force. The subscripts *i* and *j* are the Cartesian components, respectively.

For isotropic materials, the elastic properties ( bulk modulus *B*, Young’s modulus *E*, shear modulus *G*, and Poisson’s ratio ν ) of the MD simulated models must satisfy two relations [19]: G=2E/(1+ν) and B=3E/(1−2ν). Therefore, only two independent constants are required to describe the elastic behavior of isotropic material. This paper calculates Young’s modulus and Poisson’s ratio. Young’s modulus is obtained from the slope of the stress–strain curve. Moreover, Poisson’s ratio is calculated from the ratio of the lateral shrinkage in the x or z direction to the longitudinal tensile strain in the y direction.

## 3. Results and Discussion

### 3.1. Density

The different atomic ratios of all a-SiC${}_{x}$N${}_{y}$ structures and their obtained densities after quenching and relaxation at 300 K and 0 atm are shown in Table 1. The density computed by MD simulation for the a-SiC${}_{x}$N${}_{y}$ structure lies in the range of 2.247–2.831 g/cm${}^{3}$, which is in line with the X-ray photoelectron spectroscopy and MD simulation values 2.3 ± 0.3 to 3.45 ± 0.2 g/cm${}^{3}$ reported in literature [20].

### 3.2. Structural Characterization

**Figure 3:**
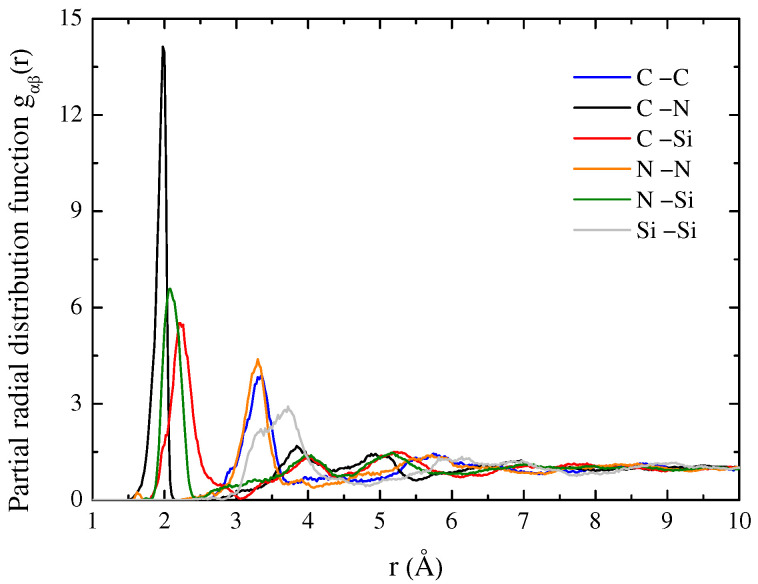
Partial radial distribution function of a-SiCN.

**Figure 4:**
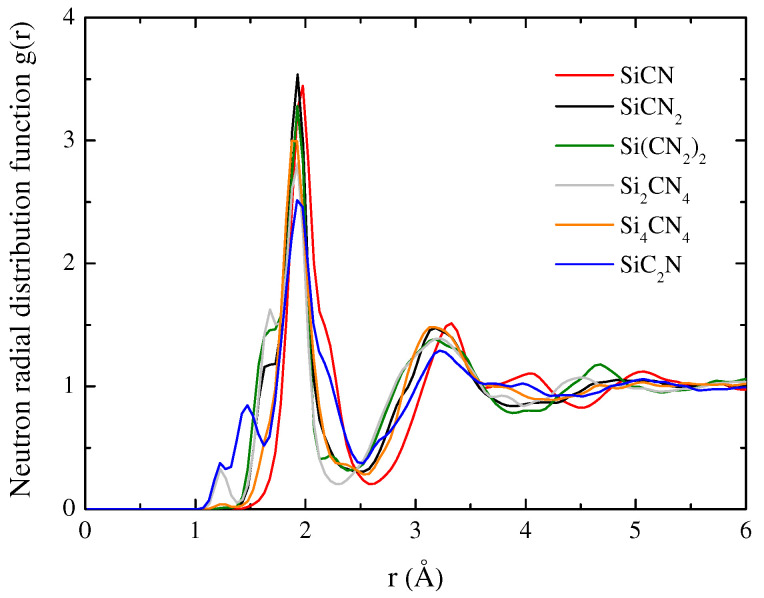
Neutron radial distribution function of a-SiC${}_{x}$N${}_{y}$.

The RDF quantifies the short-range order, while structure factors characterize the medium-range order. Figure 3 and Figure 4, respectively, shows the g${}_{\alpha \beta }$(*r*) of SiCN and g(*r*) of a-SiC${}_{x}$N${}_{y}$ structures with different atomic ratios. It shows a clear local (<3 Å) and medium-range (3–10 Å) structural ordering but lacks long-range ordering above 10 Å. The results show that the peaks of the RDF of a-SiC${}_{x}$N${}_{y}$ are gradually stabilizing after 5.5 Å, which indicates that crystallization does not occur when the a-SiC${}_{x}$N${}_{y}$ structure is cooled down at a rate of 2.5 K/ps. The RDF result shows two prominent peaks near 1.92 and 3.12 Å. The positions of the first peak are the superimposing of the bond length of several types of bonds. The first peak of all samples is attributed to the distances of C–N, Si–C, and Si–N. Furthermore, for the Si–C–N ternary amorphous structure, the peaks relate to the nearest-neighbor pair correlation of all bonds such as Si–C and Si–N are reduced, indicating that the chemical order is reduced [21].

The change in the peak height and position of the RDF reveals the structural difference between various a-SiC${}_{x}$N${}_{y}$ structures at room temperature in the glassy state. For a-SiC${}_{x}$N${}_{y}$ with different atomic ratios, the positions of the two apparent peaks are within a narrow range of 1.9 and 3.2 Å, which shows the similarity of the structure. Nevertheless, the shifting of peaks with the atomic ratio in RDFs depends on the chemical association in the amorphous structure. The shift to the right is due to the enhanced coordination of neighborhoods with larger bond lengths [22]. In particular, the different peaks of SiC${}_{2}$N appear at 1.37 and 1.43 Å, which indicates that excess carbon atoms form C–C bonds.

**Figure 5:**
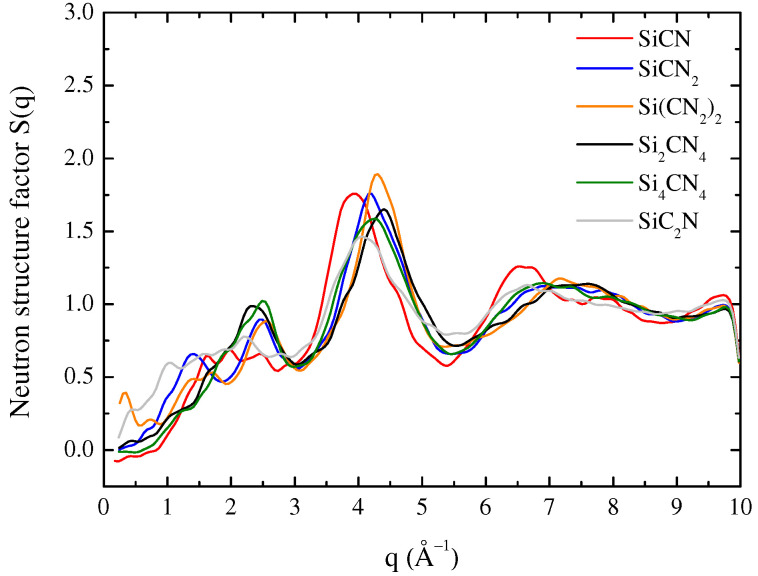
Neutron structure factor of the a-SiC${}_{x}$N${}_{y}$.

The structure is assessed by calculating the total structure factors S(q) for a-SiC${}_{x}$N${}_{y}$ with different atom content from neutron scattering, as shown in Figure 5. The distinct peaks are observed at about 2.5 and 4.2 Å, similar to the S(q) results performed by neuron wide angle scattering experiments of amorphous Si${}_{37}$C${}_{32}$N${}_{31}$ [23]. The mechanical properties are considered in the forthcoming sections based on the good agreement between the experimental and simulated structural characteristics in the density and structure factors.

### 3.3. Mechanical Properties

**Figure 6:**
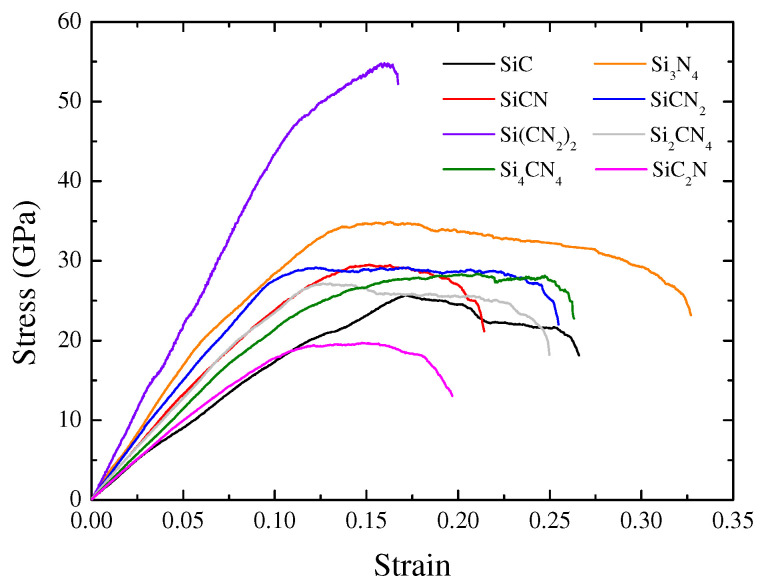
Stress–strain curves of the amorphous SiC, Si${}_{3}$N${}_{4}$, and SiC${}_{x}$N${}_{y}$.

The stress–strain curves of bulk amorphous SiC (a-SiC), Si${}_{3}$N${}_{4}$ (a-Si${}_{3}$N${}_{4}$), and a-SiC${}_{x}$N${}_{y}$ with different atomic ratios under uniaxial deformation are presented in Figure 6. The elastoplastic behavior of amorphous Si-C-N ternary materials is analyzed as follows. For a-SiC${}_{x}$N${}_{y}$, the stress–strain response first undergoes a long elastic stage, followed by a plastic deformation stage. The slope of the elastic stage is relatively constant, while the slope of the strain hardening stage gradually decreases until the stress maximum is reached. Subsequently, there is a phase of dynamic softening as the strain increases and the stress decreases slowly. Similar behavior has been reported in the plastic deformation of amorphous Si${}_{3}$N${}_{4}$ [24]. The ductility of a-SiC${}_{x}$N${}_{y}$ compounds with different atomic ratios varies greatly. In particular, a-Si(CN${}_{2}$)${}_{2}$ does not undergo a significant strain softening stage before failure.

The elastic modulus is estimated from the initial slopes of the linear portion of the tensile strain–stress curves. The linear part is from the origin to the point corresponding to a strain of 0.1. The calculated elastic moduli and Poisson’s ratios are shown in Table 2. The calculated mechanical properties of a-SiC and a-Si${}_{3}$N${}_{4}$ are compared with the available experimental data to verify the correctness of the above simulation methodology. The Young’s modulus of a-SiC is calculated to be 203.8 GPa, slightly different from the values of 232 GPa for the modulus of a-SiC deposited by PECVD at a temperature of 870 °C [25], and 240 GPa for hydrogen-free a-SiC PECVD films [26]. For a-Si${}_{3}$N${}_{4}$, our calculated modulus is 324.4 GPa. Young’s modulus of amorphous silicon nitride deposited by low-pressure chemical vapor deposition (LPCVD) is 280–290 GPa [27], while the MD calculation result is 301 GPa, corresponding to a density of 3.4 g/cm${}^{3}$ [24]. Overall, our simulated results are consistent with the experimental and other simulated data.

According to Table 2, the calculated Young’s modulus ranges from 203.80 GPa to 393.58 GPa for different compositions of a-SiC${}_{x}$N${}_{y}$. For the coating film produced by reactive magnetron sputtering, the elastic modulus decreases from 295 to 241 GPa [28] as the nitrogen content increased; or within a narrow range of around 200 GPa [29]. Whereas, the mechanical properties of amorphous thin deposited films and coatings are significantly different. For the Silicon carbon nitride films deposited in NH${}_{3}$/H${}_{2}$ mixed gas atmospheres by hot-wire chemical vapor deposition, the nano-indentation elastic modulus decreases from 170 to 120 GPa [30]. The variations in results are due to differences in chemical composition, density, film thickness, and bonding, which are affected by growth methods and parameters. The permeation of oxygen from the environment and hydrogen from the precursor into the film affects the mechanical properties [31]. The results in this paper are based on the ternary Si-C-N MD model without impurities and defects, so the calculated mechanical parameters are acceptable.

Young’s modulus of a-SiC${}_{x}$N${}_{y}$ composites with different atomic ratios do not change monotonously with the increase in carbon or nitrogen content but are related to the atomic ratio. Young’s modulus of a-Si(CN${}_{2}$)${}_{2}$ is up to 393.581 GPa, whereas Young’s modulus is only 205.512 GPa when the ratio of Si, C and N atoms is 1:2:1. In addition, Young’s modulus of other a-SiC${}_{x}$N${}_{y}$ composites is between a-SiC and a-Si${}_{3}$N${}_{4}$. By tuning the ratio of Si, C and N atoms in the growth process, the mechanical properties of a-SiC${}_{x}$N${}_{y}$ composites can be met in various applications. However, current MD models ignore growth conditions, surface effects, and possible impurities and defects, which need to be improved in further research.

## 4. Conclusions

This paper presented an MD simulation-based evaluation of the structure and mechanical properties of a-SiC${}_{x}$N${}_{y}$ films with different atomic ratios. The Si–C–N ternary amorphous model was constructed using ReaxFF potential and melt quenching method. The calculated density range of constructed a-SiC${}_{x}$N${}_{y}$ was 2.247–2.831 g/cm${}^{3}$, consistent with the experimental and MD simulated results. The peaks of structure factor for the amorphous structures were consistent with the neutron wide angle scattering experiments results, indicating a good correlation in the middle-range order of the annealing structure. On this basis, the elastic–plastic properties were analyzed through the stress–strain curve of uniaxial tension. There was an evident strain softening stage in the tensile process of a-SiC${}_{x}$N${}_{y}$, except for Si(CN${}_{2}$)${}_{2}$. The calculated Young’s modulus ranged from 206.80 GPa to 393.58 GPa, close to the experimental values of coating films produced by reactive magnetron sputtering and hydrogen-free films deposited by ion-beam sputtering deposition. Elastic properties of a-SiC${}_{x}$N${}_{y}$ were related to the atomic ratio, not just monotonically related to the carbon and nitrogen content. Overall, the atomic-scale simulation method presented here provide a reference for the chemical composition dependence of the mechanical properties of amorphous compounds. The obtained mechanical results lay the foundation for realizing multi-scale numerical simulations of reliable multi-level interconnect structures.

## Figures and Tables

**Table 1:** Densities of annealing models of a-SiC${}_{x}$N${}_{y}$.

Type	SiCN	SiCN${}_{2}$	Si(CN${}_{2}$)${}_{2}$	Si${}_{2}$CN${}_{4}$	Si${}_{4}$CN${}_{4}$	SiC${}_{2}$N
Density (g/cm${}^{3}$)	2.602	2.527	2.582	2.584	2.831	2.247

**Table 2:** Young’s modulus, and Poisson’s ratio of a-SiC${}_{x}$N${}_{y}$.

Type	Young’s Modulus (GPa)	Poisson’s Ratio
SiCN	266.874	0.412
SiCN${}_{2}$	312.837	0.405
Si(CN${}_{2}$)${}_{2}$	393.581	0.397
Si${}_{2}$CN${}_{4}$	265.628	0.391
Si${}_{4}$CN${}_{4}$	233.466	0.425
SiC${}_{2}$N	206.801	0.329

## Data Availability

The data presented in this study are available on request from the corresponding author, upon reasonable request.
